# TDM von Antidepressiva verkürzt Klinikaufenthalt: Reduktion der Aufenthaltsdauer durch routinemäßiges therapeutisches Drug-Monitoring von Antidepressiva bei Aufnahme in die Klinik

**DOI:** 10.1007/s00115-024-01636-2

**Published:** 2024-03-06

**Authors:** Maike Scherf-Clavel, Jürgen Deckert, Stefan Unterecker

**Affiliations:** Klinik und Poliklinik für Psychiatrie, Psychosomatik und Psychotherapie, Universitätsklinikum Würzburg, Margarete-Höppel-Platz 1, 97080 Würzburg, Deutschland

## Hintergrund

In der Psychiatrie ist das therapeutische Drug-Monitoring (TDM) eine Möglichkeit, personalisierte Medizin anzubieten; es ermöglicht Dosierungen individuell aufgrund der Serumkonzentration anzupassen [4].

Therapeutisches Drug-Monitoring wird vor allem für Antidepressiva mit einem hohen Empfehlungsgrad genutzt, z. B. Amitriptylin oder Clomipramin [4]. Von der AGNP (Arbeitsgemeinschaft für Neuropsychopharmakologie und Pharmakopsychiatrie)-TDM-Expertengruppe wird empfohlen, TDM u. a. dann zu nutzen, wenn die Adhärenz fraglich ist, wenn das klinische Ansprechen ausbleibt oder wenn unerwünschte Arzneimittelwirkungen unter der empfohlenen Dosierung auftreten [4]. In der deutschen S3-Leitlinie der unipolaren Depression wird TDM empfohlen, wenn der Patient/die Patientin ungenügend auf die Medikation anspricht [2].

Unser Ziel war es, zu untersuchen, ob hinsichtlich der Krankenhausaufenthaltsdauer ein Vorteil besteht, wenn TDM direkt bei Aufnahme in die Klinik angefordert wird, verglichen mit der ersten TDM-Anforderung für einen Patienten/eine Patientin erst im Verlauf des Krankenhausaufenthaltes.

## Methodik

An der Klinik und Poliklinik für Psychiatrie, Psychosomatik und Psychotherapie des Universitätsklinikums Würzburg wird seit 2003 ein routinemäßiger TDM-Service angeboten. Seit 2015 ist eine elektronische Datenbank verfügbar, in der alle TDM-Ergebnisse gesammelt werden.

Retrospektiv wurden Patient:innen mit TDM-Bestimmungen von Antidepressiva der Jahre 2015 bis 2021 ausgewertet. Einbezogen in die Analyse wurden Patient:innen mit einer depressiven Episode (F32/F33). Nicht einbezogen in die Analyse wurden Patient:innen mit einer Krankenhausaufenthaltsdauer von weniger als 7 Tagen z. B. für eine geplante Intervall-EKT-Behandlung (*n* = 39), Patient:innen, die auf eigenen Wunsch aus der Klinik entlassen wurden, Patient:innen mit einem Krankenhausaufenthalt ≥ 3 SD des Mittelwerts (*n* = 18) und Patient:innen, bei denen nur bei Entlassung ein TDM durchgeführt wurde (*n* = 130). Die Patient:innen wurden in zwei Gruppen eingeteilt, die Einteilung bezog sich auf den Zeitpunkt der ersten TDM-Anforderung: direkt bei der Aufnahme (Aufnahmedatum plus 2 Tage), oder während des Krankenhausaufenthalts in der Regel nach Umstellung der Medikation.

Die retrospektive Analyse der klinischen Routinedaten erfolgte in Übereinstimmung mit einem Votum der Würzburger Ethikkommission (20230720 03) und den Prinzipien der Deklaration von Helsinki.

### Statistische Auswertung

Die statistische Analyse wurden mit R v4.0.4 [5] durchgeführt. Unterschiede zwischen der Aufenthaltsdauer und dem Zeitpunkt der ersten TDM-Anforderung (bei Aufnahme vs. während des Krankenhausaufenthalts) wurden mithilfe des Mann-Whitney-U-Tests untersucht. Ein *p*-Wert < 0,05 wurde als signifikant angesehen. Die Berechnung der statistischen Power erfolgte mit G*Power v3.1.9.7 unter Verwendung eines zweiseitigen Post-hoc-t-Tests mit α ≤ 0,05 [3].

## Ergebnisse

Insgesamt 1067 Patient:innen kamen für die Analysen infrage. 98,31 % (*n* = 1049) der Patient:innen litten an einer schweren Depression und 1,69 % (*n* = 18) an einer mittelschweren depressiven Episode. Die durchschnittliche Dauer des Krankenhausaufenthalts betrug 47,9 Tage (Standardabweichung [SD] 25,0 Tage; Minimum 8 Tage; Maximum 142 Tage).

**Abb. 1:**
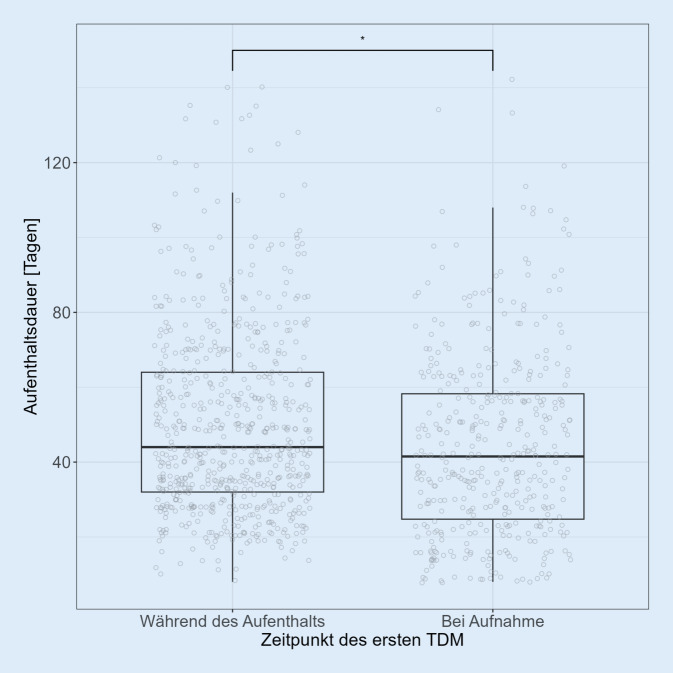
Patient:innen, bei denen therapeutisches Drug-Monitoring (TDM) direkt bei der Aufnahme angefordert wurde (*n* = 408), hatten einen signifikant kürzeren Krankenhausaufenthalt als Patient:innen, für die TDM erst während ihres Krankenhausaufenthalts angefordert wurde (*n* = 659; Mann-Whitney-U-Test; *p* = 5,84 * 10^−4^)

Patient:innen, für die TDM direkt bei der Aufnahme angefordert wurde (*n* = 408), hatten einen signifikant kürzeren Krankenhausaufenthalt (Mittelwert ± SD der Aufenthaltsdauer 44,6 ± 24,9 Tage) als Patient:innen, für die TDM erst während des Krankenhausaufenthalts angefordert wurde (*n* = 659; Mittelwert ± SD Aufenthaltsdauer 50,0 ± 24,8 Tage; *p* = 5,84 * 10^−4^; Power: 92,0 %; Abb. 1; Mittelwert ± SD Zeit bis zum ersten TDM in der Gruppe TDM während des Aufenthalts: 18,2 ± 13,0 Tage).

## Diskussion

In dieser retrospektiven Analyse wurde der Zusammenhang zwischen der Dauer des Krankenhausaufenthalts bei Patient:innen, die überwiegend (98 %) an schweren depressiven Episoden leiden, und dem Zeitpunkt der TDM-Anforderung für Antidepressiva untersucht.

Die Aufnahme vorbehandelter Patient:innen kann dabei als Ausdruck eines Nichtansprechens auf die ambulante Therapie angesehen werden und damit das TDM auch zur Aufnahme als leitliniengerecht.

Es zeigt sich eine signifikante Verkürzung der Aufenthaltsdauer, wenn TDM routinemäßig direkt bei der Aufnahme in die Klinik angefordert wurde, im Vergleich zu einer ersten TDM-Anforderung während des Krankenhausaufenthalts, unabhängig vom antidepressiven Medikament. Da die eingeschlossenen Patient:innen nur aus dem Krankenhaus entlassen wurden, wenn sie sich zumindest teilweise in Remission befanden, kann die Dauer des Krankenhausaufenthalts als Surrogatmarker für das klinische Ansprechen verwendet werden.

Eine Verkürzung der Krankenhausaufenthaltsdauer ist nicht nur für den Patienten/die Patientin, sondern auch unter wirtschaftlichen Aspekten hoch relevant. Bei täglichen Behandlungskosten von mindestens € 300 für einen Krankenhausaufenthalt lassen sich die direkten Kosten pro Patient:in um durchschnittlich € 1500 senken. Außerdem sinken die indirekten Kosten pro Patient:in durch die um 5 Tage frühere Rückkehr an den Arbeitsplatz um € 684. Indirekte Kosten wurden kalkuliert, indem das durchschnittliche monatliche Einkommen in Deutschland (€ 4105; durchschnittliches monatliches Gehalt im April 2022 [6]) verwendet wurde, um das durchschnittliche 5‑Tages-Gehalt zu berechnen. Auch wenn man nur 70 % des monatlichen Gehaltes annimmt, das von der gesetzlichen Krankenkasse bei einem Ausfall von mehr als 6 Wochen bezahlt wird [1], sinken die indirekten Kosten um € 479. In der Summe sinken die Gesamtkosten pro Patient:in um ca. € 2000. Dies vergleicht sich mit Kosten von ca. € 50 für eine TDM-Bestimmung (GOÄ-Ziffer 4210). Bei 1000 Patient:innen beläuft sich damit die Reduktion der Kosten auf bis zu 2 Mio. €.

Zusammenfassend konnten wir zum ersten Mal zeigen, dass eine frühe Anforderung von TDM schon bei der Aufnahme ins Krankenhaus die Dauer des Krankenhausaufenthalts um durchschnittlich 5 Tage verkürzt. Dies kann sowohl die Genesung der Patient:innen beschleunigen als auch die Behandlungskosten senken.

## Fazit für die Praxis

- Bei Patient:innen, die an einer Depression leiden, verkürzt eine Kontrolle der Serumkonzentrationen der Antidepressiva (TDM) routinemäßig direkt schon bei der Aufnahme ins Krankenhaus die Dauer des Krankenhausaufenthalts um durchschnittlich 5 Tage.
- Vor allem bei schweren Depressionen sollte daher direkt bei Aufnahme in die Klinik routinemäßig TDM angefordert werden, um somit die Behandlungsdauer zu verkürzen und ein schnelleres Ansprechen auf eine Medikation zu erreichen.
- Dieses Vorgehen ist dabei auch im Rahmen der aktuellen Leitlinie, da die stationäre Aufnahme bei vorbehandelten Patient:innen Ausdruck des Nichtansprechens der Therapie ist.
